# Comparison of ZnS(Ag) Scintillator and Proportional Counter Tube for Alpha Detection in Thin-Layer Chromatography

**DOI:** 10.3390/ph18010026

**Published:** 2024-12-28

**Authors:** Marc Pretze, Jan Wendrich, Holger Hartmann, Robert Freudenberg, Ralph A. Bundschuh, Jörg Kotzerke, Enrico Michler

**Affiliations:** 1 Department of Nuclear Medicine, University Hospital Carl Gustav Carus, Technical University Dresden, Fetscherstr. 74, 01307 Dresden, Germany; holger.hartmann@ukdd.de (H.H.); robert.freudenberg@ukdd.de (R.F.); ralphalexander.bundschuh@ukdd.de (R.A.B.); joerg.kotzerke@ukdd.de (J.K.); enrico.michler@ukdd.de (E.M.); 2 Eckert & Ziegler Eurotope, 13125 Berlin, Germany; jan.wendrich@ezag.de

**Keywords:** ^212^Pb, ^225^Ac, PSMA, TATE, TOC, thin-layer chromatography (TLC), quality control

## Abstract

(1) **Background**: Targeted alpha therapy is an emerging field in nuclear medicine driven by two advantages: overcoming resistance in cancer-suffering patients to beta therapies and the practical application of lower activities of ^212^Pb- and ^225^Ac-labelled peptides to achieve the same doses compared to beta therapy due to the highly cytotoxic nature of alpha particles. However, quality control of the ^212^Pb/^225^Ac-radiopharmaceuticals remains a challenge due to the low activity levels used for therapy (100 kBq/kg) and the formation of several free daughter nuclides immediately after the formulation of patient doses; (2) **Methods**: The routine alpha detection on thin-layer chromatograms (TLC) of ^212^Pb- and ^225^Ac-labelled peptides using a MiniScanPRO+ scanner combined with an alpha detector head was compared with detection using an AR-2000 scanner equipped with an open proportional counter tube. Measurement time, resolution and validity were compared for both scanners; (3) **Results**: For ^225^Ac, the quality control values of the radiochemical purity (RCP) were within the acceptance criteria 2 h after TLC development, regardless of when the TLC probe was taken. That is, if the TLC probe was taken 24 h after radiosynthesis, the true value of the RCP was not measured until 5 h after TLC development. For ^212^Pb-labelled peptides, the probe sampling did not have a high impact on the value of the RCP for the MiniScanPRO+ and AR-2000. A difference was observed when measuring TLC with the AR-2000 in different modes; (4) **Conclusions**: The MiniScanPRO+ is fast, does not require additional equipment and can also measure the gamma spectrum, which may be important for some radiopharmaceutical production sites and regulatory authorities. The AR-2000 has a better signal-to-noise ratio, and this eliminates the need for additional waiting time after TLC development.

## 1. Introduction

**Figure 1:**
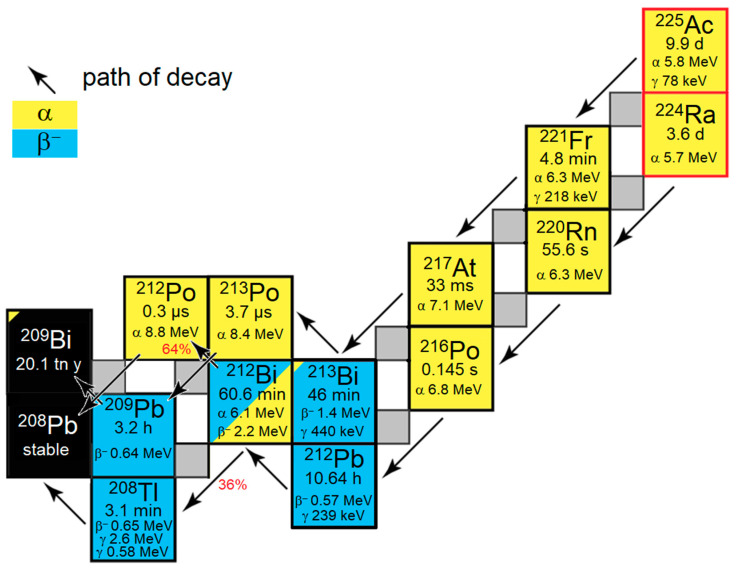
Decay diagram for the mother nuclides ^225^Ac and ^224^Ra (red square). Shown are their daughter nuclides with half-life (tn = trillion) and stable nuclides (black square), type of decay and the corresponding decay energy and important γ energies for imaging and identification in γ spectrum analyses. The arrows indicate the main decay of the nuclide. ^212^Bi has two major decays, the probability of which is given in percent (red) by the arrows.

Targeted alpha therapy (TAT) has recently come to the forefront of clinical nuclear medicine as commercial sources of alpha-emitting nuclides such as ^212^Pb (t_1/2_ = 10.6 h, Alpha particle energy Eα= 6.1 MeV (36%) and 8.8 MeV (64%)) and ^225^Ac (half-life t_1/2_ = 9.9 d, Eα = 5.8 MeV, 6.3 MeV, 7.1 MeV and 8.4 MeV) are being established [1,2]. Chelators such as TCMC (1,4,7,10-tetrakis(carbamoylmethyl)-1,4,7,10-tetraazacyclododecane) [3], lead-specific chelator (PSC) [4] and macropa (MCP) [5] have been developed. These chelators form highly stable complexes with the parent nuclides and, very importantly, with some of the daughter nuclides (Figure 1). The newly developed chelators outperform the gold standard chelator DOTA (1,4,7,10-tetraazacyclododecane-1,4,7,10-tetraacetic acid) [6] in terms of stability and labelling conditions. This has led to the clinical translation of preclinical results and the introduction of new quality control standards [4,7].

The ejection of three alpha particles from ^225^Ac in a relatively short time dramatically reduces the activity of the peptide receptor-targeted therapy by a factor of 1000 [8], and the ejection of in sum one alpha particle from ^212^Pb reduces the activity by a factor of 20–30 [9] while maintaining or even improving the therapeutic outcome. The cytotoxic nature of the alpha particles is mainly due to the destruction of Golgi, endoplasmic reticulum, mitochondria or other structures within the cell plasma rather than direct DNA double-strand breaks, as often cited in the literature [10], since the ^212^Pb- and ^225^Ac-labelled peptides certainly do not reach the cell nuclei. They are, however, able to pass the cell membrane, at least to some extent. Commercially available DOTA-conjugated peptides used for radiotherapy can be commonly labelled with ^177^Lu or ^90^Y [11] but also practically with ^225^Ac. The advantage is clear: lower activities for the patient are associated with lower doses for personnel and less shielding material. However, the inappropriate use of alpha-emitting nuclides is linked with a significantly higher dose to personnel compared to beta-emitting nuclides. The only obstacle to the wide clinical use of ^225^Ac is a lack of sources. ^225^Ac can be produced by cyclotrons using ^226^Ra targets and bombardment with protons [1]. Once this obstacle is overcome, a wide field of ^225^Ac therapy is open.

For personalised dosimetry of alpha therapy, several (un-)matched radionuclide pairs exist [12]. For therapy control and dosimetry, the three photo peaks of ^225^Ac (78 keV), ^221^Fr (218 keV) and ^213^Bi (440 keV) can be measured by SPECT [13]. In addition, the Cerenkov radiation of ^213^Bi can be used for Cerenkov luminescence imaging [14,15]. Recently, the true matched pair ^203/212^Pb came into focus via several first-in-human theranostic applications. While ^203^Pb (t_1/2_ = 52 h; 279 keV gamma ray; 81% intensity) represents an ideal elementally-matched imaging surrogate [9,16,17], ^212^Pb itself can be used for SPECT imaging [18].

A noteworthy characteristic of purified alpha-emitting nuclides mentioned in this work is that their daughter nuclides are permanently formed during radiosynthesis and quality control. They then interfere with the determination of true values for the radiochemical purity (RCP) of the radiopharmaceuticals measured by standard methods like thin-layer chromatography (TLC) and high-performance liquid chromatography (HPLC). The RCP cannot be measured by HPLC because the free daughter nuclides always show signals at the beginning of a chromatogram [7]. TLC is very well suited because the paper sheets can be set aside after development to decay the free daughter nuclides until the true RCP of the radiopharmaceuticals can be measured [19]. A second characteristic is that the purification of these radiopharmaceuticals leads to the separation of non-chelated daughter nuclides from the product and, therefore, to a different measured activity as measured by dose calibrators compared to the measured activity in an equilibrium of parent and daughter nuclides. This affects the calculated radiochemical yield (RCY), which then appears to be lower than the true RCY. Here, certain time-dependent normalisation factors for local dose calibrators are useful, but these can only be determined experimentally by comparison with dose measurements performed with a high-purity Germanium (HPGe) detector.

The MiniScanPRO+ is a scintillation counting system that mechanically moves a TLC strip under a collimated detector. During this process, the system records the count rate as a function of the position along the strip. Different types of scintillation detectors can be used with the MiniScanPRO+ to make it possible to detect alpha- (ZnS(Ag)), beta- (plastic) and gamma (NaI(Tl)) radiation. The ionising radiation interacts with the scintillator, which produces light pulses that can be detected by a photomultiplier tube. In addition, the instrument can be equipped with a multi-channel analyser (MCA) and a corresponding spectroscopy quality NaI(Tl) detector for nuclide identification.

Compared to the MiniScanPRO+, the AR-2000 is a position-sensitive gas-filled proportional counter. During the measurement, a thin wire is continuously flushed with an ionisation gas while a high voltage is applied to the wire. Ionising radiation can interact with the gas molecules to form ion pairs. Free electrons from these ion pairs are accelerated towards the anode by the electric field created by the high voltage applied to the wire. As they move, more gas molecules are ionised, creating more ion pairs. This process generates a large number of ion pairs, resulting in a detectable current pulse, which can be correlated to the position of the event along the wire. The AR-2000 allows for the separate counting of alpha and beta emissions by adjusting the operating voltage. Due to the significantly higher ionisation produced by alpha particles (more than 10 times that of beta particles), alphas can be selectively counted at a reduced high voltage setting. By reducing the high voltage to 1000 V, the AR-2000 effectively excludes beta-emitting isotopes, allowing only alpha particles to be detected. At the standard high voltage of 1500 V, both alpha and beta emissions are measured. To isolate beta emissions, alpha particles can be easily blocked by using a simple shield, such as paper or a thin layer of plastic (e.g., parafilm). As the AR-2000 uses the imaging of an entire TLC lane compared to the scanning of the MiniScanPRO+, it is possible to visualise a single TLC strip in less than a minute with almost 100 times the sensitivity of the MiniScanPRO+.

In this work, the results of two different TLC scanners (MiniScanPRO+ and AR-2000; Eckert & Ziegler, Berlin, Germany), were compared with respect to the suitability of RCP measurements of ^212^Pb- and ^225^Ac-labelled radiopharmaceuticals for clinical application.

## 2. Results

### Radiochemistry

The radiolabelling of the different precursors was performed with a >95% radiochemical yield (RCY) by using previously optimised reaction conditions [20,21]. Considering this fact, the measurement of the radiochemical purity (RCP) by TLC should only show discrepancies due to different probe development times and measurement times. The following test parameters were used:

- Amount of activity required on TLC (1–100 kBq);
- Wait time for the true RCP after TLC development immediately after synthesis;
- Wait time for the true RCP after TLC development 24 h after synthesis;
- Wait time for the true activity after synthesis and purification for calculation of the true RCY;
- Acceptance criteria: Radiochemical purity >90% right after synthesis and TLC development (prospective), >97% 2–5 h after TLC development (retrospective).

The true activity (in Becquerel) for ^225^Ac-labelled peptides was measured 2 h after the end of synthesis. The measured activity value was >95% of the true activity 1 h after synthesis. The true activity for ^212^Pb-labelled peptides was measured 4 h after synthesis. The measured activity value was >85% of the true activity 2 h after synthesis. The relatively short half-life of ^212^Pb led to significantly lower activity at the equilibrium (e.g., 10 MBq at the end of synthesis was only 7.7 MBq at equilibrium 4 h later). Therefore, the true activity of the freshly purified product should always be calculated using a normalisation factor rather than waiting for equilibrium.

**Figure 2:**
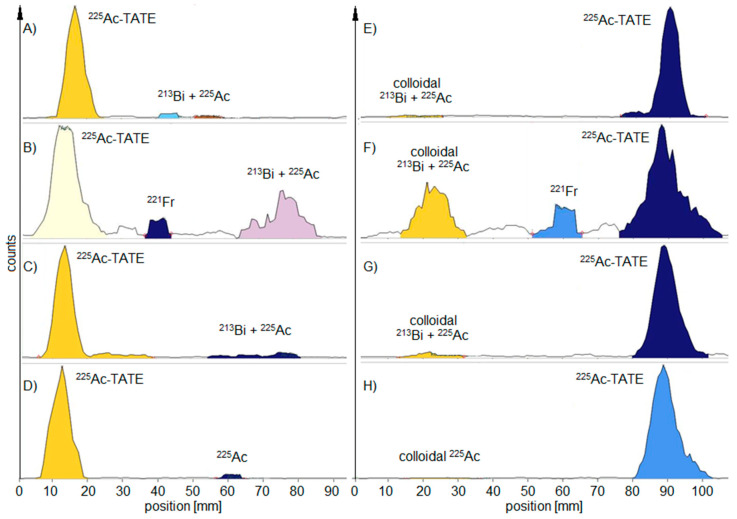
Representative radio-TLCs measured with the MiniScanPro+ at different time points: (**A**) citrate, (**E**) NH_4_Ac—TLC probe taken within 5 min after synthesis—measurement immediately after development; (**B**) citrate, (**F**) NH_4_Ac—TLC probe taken 24 h later—measurement immediately after development; (**C**) citrate, (**G**) NH_4_Ac—TLC probe taken 24 h later—measurement 2 h after development; (**D**) citrate, (**H**) NH_4_Ac—TLC probe taken 24 h later—measurement 24 h after development.

For ^225^Ac-labelled peptides, a rule for determining the true RCP using the MiniScanPRO+ was found: two hours after TLC development, the values of the RCP are within the acceptance criteria when the TLC probe was taken within the first 30 min after radiosynthesis (Table 1). If the TLC probe was taken 24 h after radiosynthesis, the true value of the RCP within the acceptance criteria was not measured until 5 h after TLC development (Figure 2).

**Figure 3:**
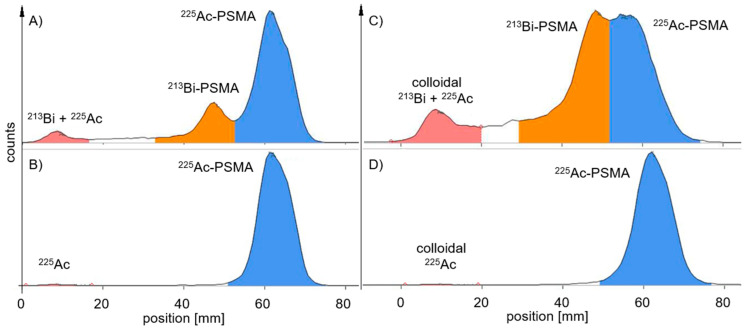
Representative radio-TLCs measured with the AR-2000 at different time points and measurement modes—probe taken immediately after synthesis: (**A**) alpha detection, 5 min after development; (**B**) alpha detection, 4 h after development (^213^Bi is decayed) (**C**) beta detection, 5 min after development—free ^213^Bi and ^213^Bi-PSMA have higher signals in beta mode (440 keV); (**D**) beta detection, 4 h after development.

With the AR-2000, the waiting time for the true RCP from a probe taken up to 24 h later was reduced to <2 h after development. Furthermore, when applying alpha detection, the AR-2000 measured RCP values within the acceptance criteria for the first 2 h of TLC probe sampling. In addition, ^213^Bi-labelled PSMA was also found on TLC with a higher signal when using beta detection compared to alpha detection measurements (Figure 3).

Five minutes after development, RCPs were < 90% for starting activities. Due to the lower signal-to-noise ratio, <10 MBq is often measured with the MiniScanPRO+. At starting activities of >10 MBq, the signal-to-noise ratio is not a continuing problem for measuring RCPs >90%. Therefore, for the MiniScanPRO+, a waiting time of >20 min after TLC development is recommended. Whereas, for the AR-2000, there is no further waiting time after TLC development for the measurement of RCPs >90%, even at starting activities of <1 MBq.

**Figure 4:**
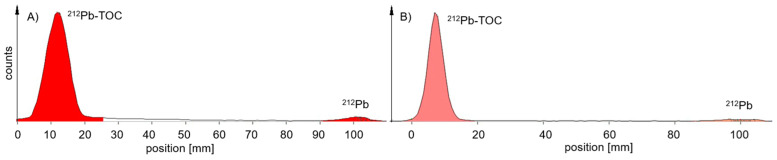
Representative radio-TLCs measured 5 min after development in citrate buffer—probe taken immediately after synthesis; (**A**) measured with the MiniScanPRO+, (**B**) measured with the AR-2000 in alpha mode, ^212^Pb-signal was lower at the front compared to measurement (**A**).

**Figure 5:**
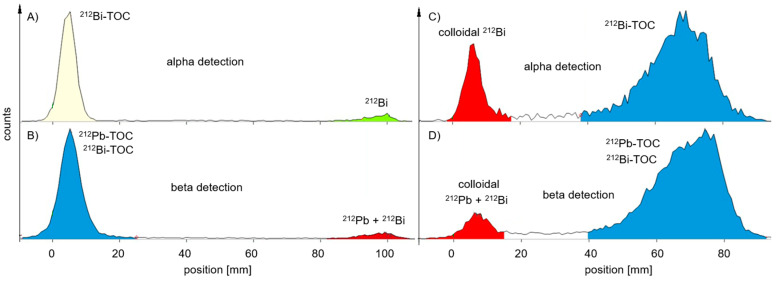
Representative radio-TLCs measured 5 min after development with AR-2000—probe taken immediately after synthesis; (**A**) TLC in citrate buffer with alpha detection—measuring 8% free ^212^Bi; (**B**) TLC in citrate buffer with beta detection—measuring 7% free ^212^Pb (238 keV) and free ^212^Bi (2.2 MeV); (**C**) TLC in NH_4_Ac with alpha detection—measuring 24% colloidal ^212^Bi; (**D**) TLC in NH_4_Ac with beta detection—measuring 8% colloidal ^212^Pb and ^212^Bi.

For ^212^Pb-labelled peptides, probe sampling did not have such an effect on the values of the RCP (Table 2) for the MiniScanPRO+ and AR-2000 (Figure 4). A difference was observed when measuring TLC with the AR-2000 in different modes. In alpha detection, when the probe was taken immediately after synthesis, values of the RCP were measured within the acceptance criteria on TLC with citrate up to 2 h after synthesis. In beta detection, when the probe was taken immediately after synthesis, values of the RCP were measured within the acceptance criteria on TLC with ammonium acetate/methanol (NH_4_Ac) up to 2 h after synthesis (Figure 5). Interestingly, on NH_4_Ac sheets, the daughter nuclide ^212^Bi showed a high signal outside the acceptance criteria in alpha detection, whereas in beta detection, the sum of free ^212^Pb and free ^212^Bi was measured, and the result was within the acceptance criteria. Therefore, it can be assumed that the out-of-specification impurity (up to 25%) in alpha detection mainly consisted of colloidal ^212^Bi, which was not efficiently chelated during the reaction (Figure 5C). However, most of the ^212^Pb can be found in the product peak when TLC is measured in beta detection (Figure 5D).

## 3. Discussion

True radiochemical purity is defined in this paper as the RCP of the product measured when all daughter nuclides are in equilibrium. Kelly et al. gave the same definition for 26 h after TLC development [19]. For ^225^Ac, however, a waiting time of one to two half-lives of ^213^Bi (46–92 min) after the TLC development is usually sufficient to obtain radiochemical purities >97%. Therefore, for safety reasons, the synthesis protocol must include two quality requirements for the RCP. In routine synthesis, the first quality requirement is an RCP >90% (assigned as prospective) because, generally, all products that reached this quality in the experiments had RCPs >97% after a 2 h waiting time and remeasurement of the TLC (Table 1). The second quality requirement is for measured product RCPs <90% (assigned as retrospective), which must be >97% RCP after 2 h. This has the following consequence for routine quality control: if the measured RCP is >90% (prospective), a second measurement is unnecessary, and the quality control can be completed. If the measured RCP is <90%, the quality control has to be extended to >2 h and the TLC has to be remeasured (retrospective) after the daughter nuclides have decayed. In most experiments, an RCP <90% was due to a later probe sampling, more than 5 min after the end of the radiosyntheses.

On the MiniScanPRO+, it was possible to determine RCPs >90% immediately after synthesis for starting activities >10 MBq. For starting activities <10 MBq, RCPs <90% were often measured with the MiniScanPRO+ within the first five minutes after TLC development because of the lower signal-to-noise ratio. Therefore, for the MiniScanPRO+ a waiting time of 20–40 min after TLC development is recommended to obtain radiochemical purities >95%. Longer waiting times of >2 h resulted in the measurement of true radiochemical purities of >97%. The AR-2000 had a 12–27 better signal-to-noise ratio for every activity tested on TLC. It was possible to determine the true radiochemical purity >97% immediately after synthesis without a waiting time after TLC development, even at starting activities of <10 MBq.

Measurement of each compound was possible with each dilution tested (10 kBq, 100 kBq) and system (MiniScanPRO+ and AR-2000). In addition, the AR-2000 produced readable spectra even at 1 kBq per TLC, whereas the MiniScanPRO+ had a disturbingly high background at this low level of activity. The optimum scan time for the MiniScanPRO+ was set at 5 mm/s. A slower scan time might produce better spectra but would not match the high sensitivity of the AR-2000.

The AR-2000 requires an ionisation gas for measurement and does not have an MCA. One bottle of this gas will last at least two years if measurements are taken twice a week. It is, therefore, very convenient to use the AR-2000 with an ionisation gas.

It is interesting to note that ^225^Ac-labelled radiopharmaceuticals did not show such high radiolysis in the final diluted and sterile solutions (Table 1, 24 h values) as speculated in the literature [22]. Of course, when the ^225^Ac nuclide decays, the ejected alpha particle or its repulsion could hit the rest of the molecule and destroy a certain percentage of it. Since the alpha particle can ejected in all possible directions around a sphere, the chance of it hitting the molecule it is attached to or molecules in solution is very small. However, when the ^225^Ac nuclide decays, it will certainly be away from the molecule. However, the long half-life of ^225^Ac means that one nuclide might remain intact for up to 9.9 days. The fact is, that the strong dilution (8–10 mL) of a very small number of radiolabelled molecules (e.g., 5 MBq ^225^Ac-PSMA-I&T consists of 50 µg or 33 nmol PSMA-I&T molecules) in the final solution leads to very low radiolysis, which was experimentally observed in this work. Therefore, the centralised production of ^225^Ac radiopharmaceuticals and distribution over 24 h might be possible. With a waiting time of up to 5 h for determination of the true RCP by TLC after 24 h, the ^225^Ac radiopharmaceuticals could also be checked for quality on arrival at the clinic in the early morning for administration to patients during the day. In addition, new chelators, such as PSC [4] and MCP [7], are known to bind to the respective radionuclides even at room temperature. Therefore, a certain safety level of unlabelled precursor in the final solution could lead to a rechelation of daughter nuclides like ^212^Bi (to PSC) or ^213^Bi (to MCP). It would be interesting to know whether a high dilution of the final solution interferes with the theoretical rechelation process. However, a lower dilution could lead to a higher radiolysis. The optimum volume for low radiolysis and a high rechelation rate in the final solution will be investigated experimentally in the near future.

For ^225^Ac-labelled radiopharmaceuticals, DTPA may be added to avoid a high proportion of free daughter nuclides like ^213^Bi in the final solution, especially if the quality control is extended to >2 h by the above procedure [8]. The bound ^213^Bi-DTPA may pass through the kidneys more rapidly than free ^213^Bi, which physiologically binds to the kidneys [23]. Without DTPA in the final solution, most patients in our clinic have shown good renal function up to the fourth cycle of ^225^Ac. However, some patients develop reduced renal function before the fourth cycle of ^225^Ac-treatment, and adding DTPA to the final solution may prolong good renal function in these patients. The literature suggests that 0.1% DTPA should be in the final solution [8], which can be added either to the diluent during automated syntheses [20] or directly to the final vial, which may be the better choice. However, DTPA in the diluent could act as a scavenger for free ^225^Ac, chelating free ^225^Ac from the purification cartridge and flushing it as ^225^Ac-DTPA into the product solution. TLC quality control would then show more ^225^Ac in the non-product fraction, resulting in a false true RCP and a complicated situation.

**Figure 6:**
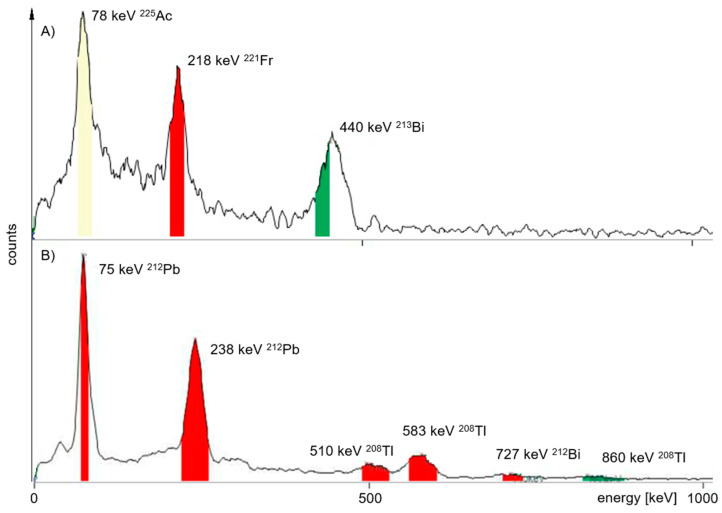
Respective gamma spectra measured with the MCA of the MiniScanPro+ for verification of the radio nuclidic purity. Gamma energy found given in keV (percentage of probability): (**A**) ^225^Ac: 78 keV (3%), ^221^Fr: 218 keV (12%), ^213^Bi: 440 keV (26%); (**B**) ^212^Pb: 75, 238 keV; ^208^Tl: 510 (22.6%), 583 (85.0%), 860 (12.5%) keV; ^212^Bi: 727 (6.7%) keV.

The MiniScanPRO+ has the ability to measure a gamma spectrum using a spectroscopic grade NaI(Tl) detector (Figure 6). This characteristic measurement is important when radionuclide purity needs to be measured for product approval. The sensitivity of the MCA is sufficient to discriminate between the important γ signals of ^212^Pb and ^225^Ac, but in fact, this test is unnecessary for quality control in most cases since the radionuclide purity is also given by the certificate of quality and purity provided by the distributors of the radionuclides.

Theoretically, the spectra of ^225^Ac peptides measured at the start of the TLC (e.g., a high percentage of ^225^Ac-TATE only when developed with citrate) and at the front of the TLC (e.g., a low percentage of free ^225^Ac and high percentage of free ^221^Fr + ^213^Bi) could differ in the shape and intensity of the individual gamma signals at 78 keV (^225^Ac), 218 keV (^221^Fr) and 440 keV (^213^Bi). Experimentally, no difference in the peak height of the signals between the start and the front of the TLC was found, even at a high activity in the range of ~37 MBq, since the difference between bound and free ^225^Ac should be the greatest. Therefore, with the MCA of the MiniScanPro+, it was not possible to bypass the waiting time for the determination of the true RCP by performing calculations on the percentage of the product peak using a certain nuclide quotient obtained from the gamma spectrum. In this case, only an HPGe may have sufficient sensitivity to detect differences in the peak height of the γ signals between the start and front fractions of the TLCs.

To date, the measurement of radionuclide purity has only been required by regulatory authorities for the production of beta-emitting radiopharmaceuticals. To date, there is no such monography for alpha-emitting radiopharmaceuticals, but this is expected to change in the next few years as the use of alpha-emitting radiopharmaceuticals in the clinic increases and companies are already working on approvals for such alpha-emitting radiopharmaceuticals. An example of beta-emitting radionuclides is ^177^Lu, which requires approval at >99.9% radionuclide purity, as ^177m^Lu is an important impurity in the production of ^177^Lu and should not exceed 0.1% (Ph. Eur. 11.0 07/2017:2798 corrected 9.3).

## 4. Materials and Methods

All reagents and solvents were purchased in the highest purity from commercial suppliers and were used without further purification. PSC-PEG_2_-TOC (VMT-α-NET) and ^224^Ra/^212^Pb-generator (VMT-α-GEN) were obtained from Perspective Therapeutics Inc., Coralville, IA, USA. ^225^Ac was obtained from van Overeem nuclear bv (Breda, The Netherlands). The RCP was monitored by thin-layer chromatography (TLC) on iTLC-SG plates (Agilent, Santa Clara, CA, USA).

Measurement of the radio nuclide purity (RNP) and evaluation of the radio-TLC were performed with a thin-layer scanner (MiniScanPRO+, Eckert & Ziegler Eurotope GmbH, Berlin, Germany) equipped with a Model 43-2 alpha detector ZnS(Ag) scintillator (Ludlum Measurements, Sweetwater, TX, USA) and a built-in multi-channel analyser (MCA) for gamma spectroscopy or with a proportional gas-counter (AR-2000, Eckert & Ziegler Eurotope GmbH, Berlin, Germany) equipped with an ionisation gas (90% Ar/10% CH_4_ or 90% Ar/10% CO_2_). CORGON-10 was purchased from Linde Gas (Dresden, Germany). The operator has to choose between Ar/CO_2_ (90/10), a commonly used welding gas, or Ar/CH_4_ (90/10), widely known as P10 gas. The gas flow during the operation was about 2–3 L/min. With measurement times of about 1 min per strip, this is enough for more than 2000 measurements with a 20 L 200 bar bottle of counting gas and adds less than 10 c per measurement to production costs.

Radio-HPLC was performed on a Shimadzu HPLC system (Thermo Scientific, Dreieich, Germany), equipped with a reverse phase column (Analytical: Merck Chromolith RP-18e; 100 × 4.6 mm plus a guard column 5 × 4.6 mm and a UV-diode array detector (220 nm). The solvent system used was a gradient of acetonitrile:water (containing 0.05% TFA) (0–13 min: 0–60% MeCN) at a flow rate of 1.6 mL/min unless otherwise stated. The pH was measured with a reflection photometer (QUANTOFIX Relax, Macherey-Nagel GmbH & Co. KG, Düren, Germany).

### 4.1. Radiochemistry

Automatic ^225^Ac-radiolabelling of DOTA-TATE or DOTAGA-PSMA-I&T was performed according to an already-established protocol [20]. Briefly, solid ^225^Ac(NO_3_)_3_ was dissolved in 100–500 µL of 0.1 M HCl_suprapure_. A 10–20 µg/MBq precursor in H_2_O_suprapure_ (1 µg/µL) was added to a 5 mL conical vial together with 2 mL 0.3 M NaAc/AcOH buffer (pH 5.7, 99.99% trace metal). The reaction was performed at 105°C for 35 min. Purification was performed using a CM cartridge (WAT023531).

Automatic ^212^Pb-radiolabelling of PSC-PEG_2_-TOC was performed according to an established protocol [24]. In brief, 0.1 µg/MBq of precursor in H_2_O_suprapure_ (1 µg/µL) was added to a 5 mL conical vial together with 100 µL EtOH_absolute_, 290 µL 1 M NaAc/AcOH buffer (pH 4, 99,99% trace metal) and 2 mg sodium ascorbate (Ph. Eur.) in 100 µL H_2_O_suprapur_. The reaction was performed at 105°C for 60 min. At this condition, the daughter nuclide ^212^Bi is also chelated at >80%. Purification was performed using a C18 cartridge (WAT023501).

### 4.2. Thin-Layer Chromatography

- RCP was determined by TLC either by cutting the developed TLC into two pieces and measuring their activity using a surface contamination monitor (CoMo-170, Nuvia Instruments, Dresden, Germany) or by a TLC scanner;
- For the detection of colloidal ^225^Ac hydroxide, TLC was performed in 1 M NH_4_Ac:MeOH 1:1 on silica gel-aluminum sheets for ^225^Ac-PSMA-I&T and on ITLC-SG for ^225^Ac-TATE;
- For the detection of free ^225^Ac, TLC was performed on silica gel-aluminum sheets in 0.1 M citrate buffer (pH 5.0);
- For the detection of colloidal ^212^Pb hydroxide, TLC was performed in 1 M NH_4_Ac:MeOH 1:1 on ITLC-SG;
- For the detection of free ^212^Pb, TLC was performed on ITLC-SG in 0.1 M citrate buffer (pH 5.0);
- RNP and exact volume activity were determined using an HPGe detector (Canberra GmbH, Rüsselsheim, Germany);
- For endotoxin level determination, 10 µL of the product solution was diluted with 990 µL sterile water (1:100) using an EndoSafe PTS (Charles River, Sulzfeld, Germany);
- The pH was determined using a reflection photometer.

Both scanners used the same RaPET chromatography software and were handled similarly. Throughout experimental measurements, no issues were observed. However, in routine measurements, some minor problems were registered in the software or communication between the scanner and the computer. In most cases, this was due to incorrect handling by personnel. The maintenance is thought to be undertaken every 1–2 years by calibration sources containing ^22^Na for MiniScanPRO+ and ^14^C for the AR-2000. Actually, ^225^Ac or ^212^Pb in an equilibrium state might be used for the calibration of the MiniScanPRO+ because of the mixture of daughter nuclides with different gamma energies. The scanners were used for over three years without running into maintenance issues. For the AR-2000, the measuring wire in the counting head can be seen as consumable, which might need to be changed on a regular basis depending on handling by the operator and the number of samples as well as the amount of activity. Anyhow, changing the wire is an easy and straightforward process, which can be completed within minutes by the operator and does not require any external service personnel.

## 5. Conclusions

Two TLC scanners were compared for their ability to measure the RCP of radiopharmaceuticals radiolabelled with ^212^Pb and ^225^Ac. Both scanners have their advantages and disadvantages. The MiniScanPRO+ is fast, does not require additional equipment and can also measure the gamma spectrum, which may be important for some radiopharmaceutical production sites and regulatory authorities. Unfortunately, measurements of the gamma spectrum revealed no difference in the peak area between high and low percentages of ^225^Ac in the individual spots at the start and front of the TLC, presumably due to the low sensitivity of the MCA.

The AR-2000 requires an ionisation gas to operate. With independent alpha and beta detection, it was possible to produce clearer TLCs with a better signal-to-noise ratio than the MiniScanPRO+. This eliminated the need for an additional waiting time after TLC development. Quality control may be faster than with the MiniScanPRO+ if the waiting time for probe sampling is longer than five minutes after radiosynthesis. However, the AR-2000 cannot measure a gamma spectrum, but as mentioned above, this additional measurement may not be required by regulatory authorities as the radionuclides themselves already have a certificate of quality and purity when they are delivered.

In our opinion, the best quality control setup would consist of both scanners as they support each other. Otherwise, the decision depends on the local regulatory authorities and the head of quality control, who have to consider which scanner would better fit into a safe routine quality control of alpha radiopharmaceuticals.

## Figures and Tables

**Table 1:** Comparison of TLC measurements for determining radiochemical purity of ^225^Ac peptides.

TLC Development	TLC Measurement	Product Peak (%) MiniScanPRO+				Product Peak (%) AR-2000			
		^225^Ac-PSMA-I&T		^225^Ac-TATE		^225^Ac-PSMA-I&T		^225^Ac-TATE	
		Citrate	NH_4_Az	Citrate	NH_4_Az	Citrate	NH_4_Az	Citrate	NH_4_Az
Immediately after synthesis	immediately	94.7	90.4	94.1	96.6	97.6	95.5	99.6	99.2
	2 h later	98.1	99.2	n.d. *	n.d.	99.9	99.5	n.d.	n.d.
24 h after synthesis	immediately	55.7	85.7	66.9	75.6	82.9	84.8	86.4	77.8
	2 h later	91.3	94.3	90.0	94.8	96.5	94.3	96.5	97.5
	5 h later	98.1	96.6	n.d.	n.d.	98.4	96.1	n.d.	n.d.
	24 h later	98.7	97.5	96.7	98.9	98.4	96.3	98.9	99.4

* n.d. = not determined.

**Table 2:** Comparison of TLC measurements for determining the radiochemical purity of ^212^Pb peptides.

TLC Development	TLC Measurement Time	Product Peak [%] MiniScanPRO+		Product Peak [%] AR-2000	
		^212^Pb-VMT-α-NET		^212^Pb-VMT-α-NET	
		Citrate	NH_4_Az	Citrate	NH_4_Az
entry	immediately	95.5	96.9	93.1	94.7
	6 h later	95.3	94.8	96.5	90.0
	18 h later	95.8	96.8	96.7	94.7

## Data Availability

All data can be referred to on request to the corresponding author.

## References

[1] Apostolidis C., Molinet R., McGinley J., Abbas K., Mollenbeck J., Morgenstern A. (2005). Cyclotron production of Ac-225 for targeted alpha therapy. Appl. Radiat. Isot..

[2] Pruszyński M., Walczak R., Rodak M., Bruchertseifer F., Morgenstern A., Bilewicz A. (2021). Radiochemical separation of ^224^Ra from ^232^U and ^228^Th sources for ^224^Ra/^212^Pb/^212^Bi generator. Appl. Radiat. Isot..

[3] Dos Santos J.C., Schäfer M., Bauder-Wüst U., Lehnert W., Leotta K., Morgenstern A., Kopka K., Haberkorn U., Mier W., Kratochwil C. (2019). Development and dosimetry of ^203^Pb/^212^Pb-labelled PSMA ligands: Bringing "the lead" into PSMA-targeted alpha therapy?. Eur. J. Nucl. Med. Mol. Imaging.

[4] Li M., Baumhover N.J., Liu D., Cagle B.S., Boschetti F., Paulin G., Lee D., Dai Z., Obot E.R., Marks B.M. (2023). Preclinical evaluation of a lead specific chelator (PSC) conjugated to radiopeptides for ^203^Pb and ^212^Pb-Based theranostics. Pharmaceutics.

[5] Thiele N.A., Brown V., Kelly J.M., Amor-Coarasa A., Jermilova U., MacMillan S.N., Nikolopoulou A., Ponnala S., Ramogida C.F., Robertson A.K.H. (2017). An Eighteen-Membered Macrocyclic Ligand for Actinium-225 Targeted Alpha Therapy. Angew. Chem. Int. Ed. Engl..

[6] Li M., Zhang X., Quinn T.P., Lee D., Liu D., Kunkel F., Zimmerman B.E., McAlister D., Olewein K.R., Menda Y. (2017). Automated cassette-based production of high specific activity [^203/212^Pb] peptide-based theranostic radiopharmaceuticals for image-guided radionuclide therapy for cancer. Appl. Radiat. Isot..

[7] Reissig F., Bauer D., Zarschler K., Novy Z., Bendova K., Ludik M.C., Kopka K., Pietzsch H.J., Petrik M., Mamat C. (2021). Towards targeted alpha therapy with Actinium-225: Chelators for mild condition radiolabeling and targeting PSMA—A proof of concept study. Cancers.

[8] Kratochwil C., Bruchertseifer F., Giesel F.L., Weis M., Verburg F.A., Mottaghy F., Kopka K., Apostolidis C., Haberkorn U., Morgenstern A. (2016). ^225^Ac-PSMA-617 for PSMA-targeted α-radiation therapy of metastatic castration-resistant prostate cancer. J. Nucl. Med..

[9] Delpassand E.S., Tworowska I., Esfandiari R., Torgue J., Hurt J., Shafie A., Núnez R. (2022). Targeted α-emitter therapy with ^212^Pb-DOTAMTATE for the treatment of metastatic SSTR-expressing neuroendocrine tumors: First-in-humans dose-escalation clinical trial. J. Nucl. Med..

[10] Graf F., Fahrer J., Maus S., Morgenstern A., Bruchertseifer F., Venkatachalam S., Fottner C., Weber M.M., Huelsenbeck J., Schreckenberger M. (2014). DNA double strand breaks as predictor of efficacy of the alpha-particle emitter Ac-225 and the electron emitter Lu-177 for somatostatin receptor targeted radiotherapy. PLoS ONE.

[11] Roohi S., Rizvi S.K., Naqvi S.A.R. (2021). ^177^Lu-DOTATATE Peptide Receptor Radionuclide Therapy: Indigenously Developed Freeze Dried Cold Kit and Biological Response in In-Vitro and In-Vivo Models. Dose Response.

[12] Nelson B.J.B., Wilson J., Andersson J.D., Wuest F. (2023). Theranostic Imaging Surrogates for Targeted Alpha Therapy: Progress in Production, Purification, and Applications. Pharmaceuticals.

[13] Usmani S., Rasheed R., Al Kandari F., Marafi F., Naqvi S.A.R. (2019). ^225^Ac prostate-specific membrane antigen posttherapy α imaging: Comparing 2 and 3 photopeaks. Clin. Nucl. Med..

[14] Pandya D.N., Hantgan R., Budzevich M.M., Kock N.D., Morse D.L., Batista I., Mintz A., Li K.C., Wadas T.J. (2016). Preliminary Therapy Evaluation of (225)Ac-DOTA-c(RGDyK) Demonstrates that Cerenkov Radiation Derived from (225)Ac Daughter Decay Can Be Detected by Optical Imaging for In Vivo Tumor Visualization. Theranostics.

[15] Beattie B.J., Thorek D.L., Schmidtlein C.R., Pentlow K.S., Humm J.L., Hielscher A.H. (2012). Quantitative modeling of Cerenkov light production efficiency from medical radionuclides. PLoS ONE.

[16] Meredith R., Torgue J., Shen S., Fisher D.R., Banaga E., Bunch P., Morgan D., Fan J., Straughn J.M. (2014). Dose escalation and dosimetry of first-in-human alpha radioimmunotherapy with ^212^Pb-TCMC-trastuzumab. J. Nucl. Med.

[17] Müller D., Herrmann H., Schultz M.K., Solbach C., Ettrich T., Prasad V. (2023). ^203^Pb-VMT-α-NET scintigraphy of a patient with neuroendocrine tumor. Clin. Nucl. Med..

[18] Mikalsen L.T.G., Kvassheim M., Stokke C. (2023). Optimized SPECT Imaging of ^224^Ra α-particle therapy by ^212^Pb photon emissions. J. Nucl. Med.

[19] Kelly J.M., Amor-Coarasa A., Sweeney E., Wilson J.J., Causey P.W., Babich J.W. (2021). A suitable time point for quantifying the radiochemical purity of ^225^Ac-labeled radiopharmaceuticals. EJNMMI Radiopharm. Chem..

[20] Pretze M., Kunkel F., Runge R., Freudenberg R., Braune A., Hartmann H., Schwarz U., Brogsitter C., Kotzerke J. (2021). Ac-EAZY! Towards GMP-compliant module syntheses of ^225^Ac-labeled peptides for clinical application. Pharmaceuticals.

[21] Pretze M., Michler E., Runge R., Wetzig K., Tietze K., Brandt F., Schultz M.K., Kotzerke J. (2023). Influence of the Molar Activity of ^203/212^Pb-PSC-PEG_2_-TOC on Somatostatin Receptor Type 2-Binding and Cell Uptake. Pharmaceuticals.

[22] Hamisu A., Khiter O., Al-Zhrani S., Haridh W.S.B., Al-Hadeethi Y., Sayyed M.I., Tijani S.A. (2024). The use of nanomaterial polymeric materials as ionizing radiation shields. Radiat. Phys. Chem..

[23] Essler M., Gartner F.C., Neff F., Blechert B., Senekowitsch-Schmidtke R., Bruchertseifer F., Morgenstern A., Seidl C. (2012). Therapeutic efficacy and toxicity of 225Ac-labelled vs. 213Bi-labelled tumour-homing peptides in a preclinical mouse model of peritoneal carcinomatosis. Eur. J. Nucl. Med. Mol. Imaging.

[24] Pretze M., Michler E., Kästner D., Kunkel F., Sagastume E.A., Schultz M.K., Kotzerke J. (2024). Lead-it-EAZY! GMP-compliant production of [^212^Pb]Pb-PSC-PEG_2_-TOC. EJNMMI Radiopharm. Chem..

